# Ubiquitylation and endocytosis of the human LAT1/SLC7A5 amino acid transporter

**DOI:** 10.1038/s41598-019-53065-w

**Published:** 2019-11-14

**Authors:** Céline Barthelemy, Bruno André

**Affiliations:** Molecular Physiology of the Cell, Université libre de Bruxelles (ULB), IBMM (Biopark), Gosselies, Belgium

**Keywords:** Endocytosis, Ubiquitylation

## Abstract

The human L-type amino acid transporter 1 (LAT1), also known as SLC7A5, catalyzes the transport of large neutral amino acids across the plasma membrane. As the main transporter of several essential amino acids, notably leucine, LAT1 plays an important role in mTORC1 activation. Furthermore, it is overexpressed in various types of cancer cells, where it contributes importantly to sustained growth. Despite the importance of LAT1 in normal and tumor cells, little is known about the mechanisms that might control its activity, for example by promoting its downregulation via endocytosis. Here we report that in HeLa cells, activation of protein kinase C by phorbol 12-myristate 13-acetate (PMA) triggers efficient endocytosis and degradation of LAT1. Under these conditions we found LAT1 downregulation to correlate with increased LAT1 ubiquitylation. This modification was considerably reduced in cells depleted of the Nedd4-2 ubiquitin ligase. By systematically mutagenizing the residues of the LAT1 cytosolic tails, we identified a group of three close lysines (K19, K25, K30) in the N-terminal tail that are important for PMA-induced ubiquitylation and downregulation. Our study thus unravels a mechanism of induced endocytosis of LAT1 elicited by Nedd4-2-mediated ubiquitylation of the transporter’s N-terminal tail.

## Introduction

Regulation of plasma membrane nutrient transporters is crucial for cell homeostasis. A common inhibition mechanism of these proteins involves their removal from the cell surface by selective sorting into endocytosis vesicles. Once internalized, the transporters can potentially progress along the endocytic pathway and be delivered to the lysosome, where they are degraded. This downregulation mechanism has been particularly well studied in yeast, where ubiquitin (Ub) is the signal that generally triggers transporter endocytosis^1–4^. This ubiquitylation is catalyzed by the Rsp5/Npi1 ubiquitin ligase, which contains a C2 domain, three WW domains, and a C-terminal catalytic domain (HECT)^5–7^. The WW domains typically bind to PY motifs exposed by the target proteins or α-arrestin-like adaptors for Rsp5 interacting with them^8,9^.

In mammalian cells also, Ub plays an important role in downregulating multiple plasma membrane transporters and channels^10^. This was initially illustrated by the epithelial Na^+^ channel (ENaC) in the context of the study of Liddle’s syndrome, a hereditary form of hypertension^11^. ENaC ubiquitylation involves the Nedd4-2 Ub ligase, which binds directly to PY motifs present on ENaC subunits^8^. Nedd4-2 is a homolog of yeast Rsp5 and one of nine members of the Nedd4 family of HECT Ub ligases^9^. Nedd4-type Ub ligases have since been shown to promote Ub-dependent downregulation of multiple transporters, including the dopamine transporter (DAT)^12^, the glutamate transporter 1 (GLT-1)^13^, the iron transporter (DMT1)^14^, the sodium-coupled neutral amino acid transporter 3 (SNAT3)^15^, and the cationic amino acid transporter (CAT1)^16^. Transporter endocytosis is often elicited by addition of PMA (phorbol 12‐myristate 13‐acetate), an activator of protein kinase C (PKC). The mammalian counterparts of the yeast α-arrestins are the ARRestin Domain Containing (ARRDC) proteins, one of which is reported to promote endocytosis of the GLUT1 and GLUT4 glucose transporters^17,18^.

LAT1 (L-Type amino acid transporter 1) is a bidirectional transporter of large neutral amino acids (Leu, Val, Ile, Phe, Trp, His, Met, Tyr)^19–22^. As one of the main transporters of several essential amino acids including leucine, LAT1 plays an important role in activating the mTORC1 (mechanistic Target of Rapamycin Complex 1) kinase complex^23–28^. Besides the important role of LAT1 in mTORC1 control under normal physiological conditions, for instance during T cell activation^29^, LAT1 is also important in sustaining the high metabolic demands and rapid proliferation of tumor cells^22,26,30^. Moreover, overexpressed LAT1 is a negative prognostic factor in various types of cancer, such as glioma^31^, renal cell carcinoma^32^, prostate cancer^33^ and breast cancer^34^. LAT1/SLC7A5 is a member of the SLC7 solute carrier family, which comprises two subfamilies: the cationic amino acid transporters (CATs, SLC7A1-4) and the L-type amino acid transporters (LATs, SLC7A5-11)^35^. LAT1 is associated, via a disulfide bridge, with the 4F2hc type II membrane glycoprotein, and this linkage is essential to the proper transport of LAT1 and its localization to the plasma membrane^22,36^. Recently, the tertiary structure of the human LAT1-4F2hc complex was solved by cryo-electron microscopy^37^. In agreement with prior predictions^38–40^, the 12 transmembrane segments of LAT1 were found organized in a canonical LeuT fold^37^.

The intracellular trafficking of LAT1, and notably the mechanisms promoting its endocytosis, remain poorly known. In a recent study, we isolated a HeLa cell line stably expressing a LAT1-GFP construct. In these cells, we found LAT1 to undergo endocytosis in response to FTY720^41^, a sphingoid base analog that acts as an anticancer agent in animal models^42^. We also obtained evidence that this endocytosis results from inhibition of nutrient transport and mTORC1 inhibition, and that a similar mechanism accounts for FTY720-induced ubiquitylation and endocytosis of multiple transporters in yeast^41^. We now report that PKC activation by PMA induces rapid endocytosis and degradation of LAT1, that this downregulation coincides with increased ubiquitylation of LAT1, and that this modification involves the Nedd4-2 Ub ligase. We have also identified in the N-terminal tail of LAT1 three close lysines which, when mutated, confer protection against PMA-induced ubiquitylation and downregulation. Our study thus shows that induced ubiquitylation of LAT1, involving Nedd4-2, promotes its endocytosis. This could have important implications for future work on the role of LAT1 in normal and tumor cells.

## Results

### LAT1 is internalized and degraded in response to PMA

**Figure 1:**
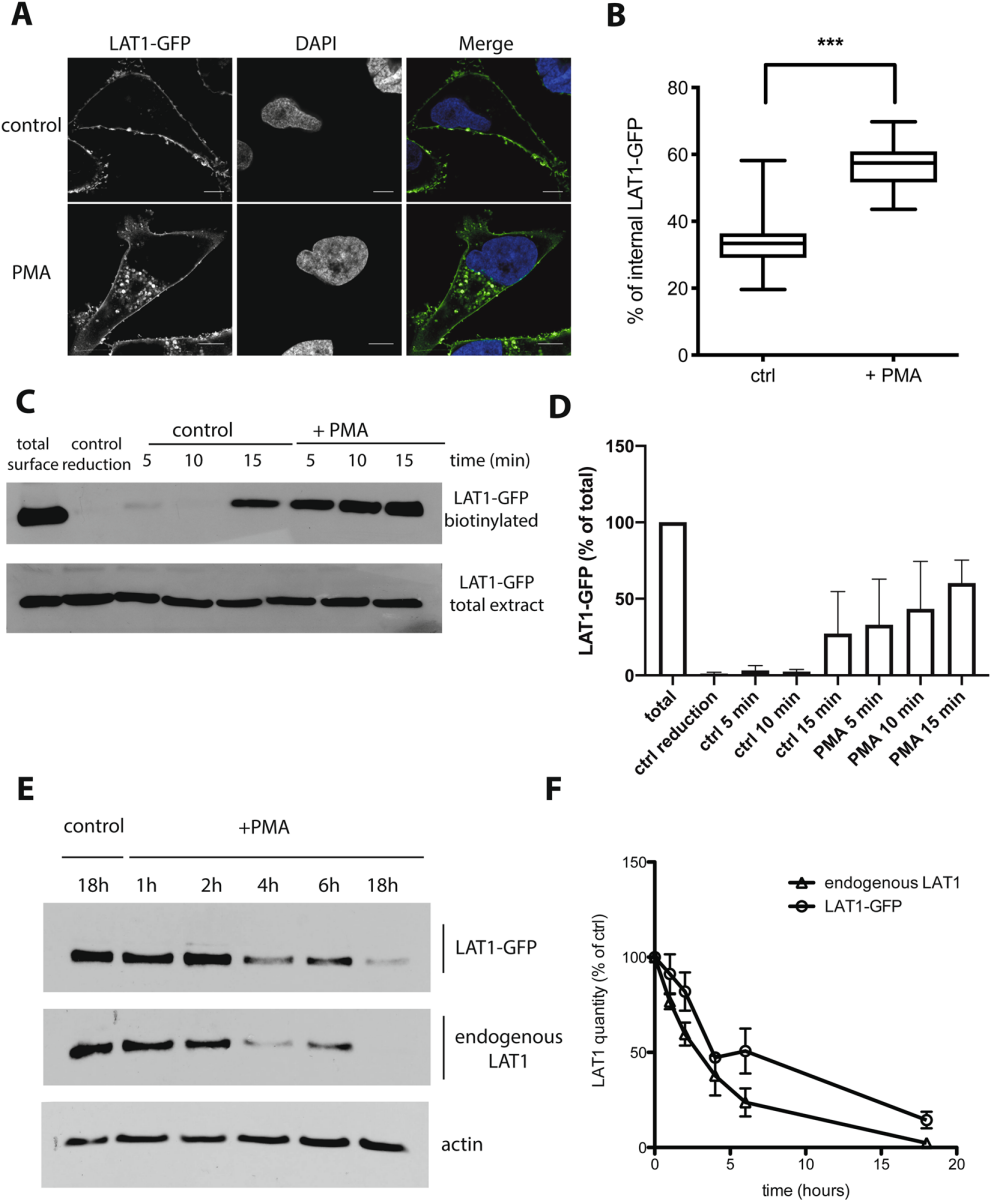
LAT1 is internalized and degraded in response to PMA. **(A)** Cells of the stable T-REx HeLa line expressing LAT1-GFP were incubated with 25 mM (NH_4_)_2_SO_4_ for 18 h and then incubated in the presence of DMSO (control) or PMA (1 µM) for 4 h. The cells were fixed and the localization of LAT1-GFP was examined by confocal microscopy. **(B)** Quantification of PMA-induced LAT1-GFP internalization. The fluorescence signals of at least 60 cells from three independent experiments were quantified. The graph shows the percentage of intracellular immunofluorescence determined as detailed under Methods. Unpaired t-test, ***p < 0.001. Scale bars, 5 μm. **(C)** Cells of the stable T-REx HeLa line expressing LAT1-GFP were incubated with sulfo-NHS-biotin for 30 min at 4 °C. The cells were then incubated in the presence of DMSO (control) or PMA (1 µM final concentration) for the indicated times at either 4 °C, to block membrane trafficking (control reduction), or 37 °C. The cells were washed and the remaining surface biotin was then cleaved off except in a control sample (total surface). The biotinylated proteins were purified by affinity chromatography with streptavidin-coated beads. LAT1-GFP was then detected by immunoblotting with anti-GFP antibody. A blot representative of three independent experiments is shown. **(D)** Quantification of the immunodetected signals in three independent experiments performed as in C. Data are presented as percentages of total surface and error bars represent standard deviations. **(E)** Cells of the stable T-REx HeLa line expressing LAT1-GFP were incubated with DMSO (control) or 1 μM PMA for the indicated times. Cell extracts were immunoblotted with anti-LAT1 antibody, to detect endogenous LAT1 and LAT1-GFP, and with anti-actin antibody. **(F)** Quantification of the signals in immunoblots performed as in E. The graph shows the mean of three independent experiments. The error bars represent standard deviations.

We previously generated a stable HeLa T-Rex cell line expressing LAT1-GFP under the control of a tetracycline-regulated promoter^41^. In the present work, these cells were first grown in the presence of tetracycline for 24 h to induce LAT1-GFP synthesis, and then transferred for 40 h to a medium without the antibiotic. Under these conditions, the induced LAT1-GFP localized mainly to the plasma membrane (Fig. 1A)^41^. We then sought to define conditions for studying the mechanisms of LAT1 endocytosis. We had previously observed that addition of FTY720 triggers redistribution of cell-surface LAT1-GFP into early endosomes, but this process was partial and relatively slow^41^. Furthermore, FTY720 appears toxic to cells upon prolonged incubation. We thus tested other conditions, including addition of phorbol 12‐myristate 13‐acetate (PMA), a PKC-stimulating compound shown to induce the endocytosis of several plasma membrane transporters^13,16,43–45^. Cells were incubated with NH_4_^+^ to inhibit lysosomal proteolysis and then treated with PMA or its solvent (DMSO). By confocal microscopy, we observed that PMA did cause significant internalization of cell-surface LAT1-GFP (Fig. 1A,B). This effect of PMA was confirmed when the cells were pretreated with a cell-impermeable agent causing biotinylation of surface proteins. After PMA treatment, the remaining cell-surface biotin was cleaved off, and the internalized biotinylated proteins were purified on streptavidin beads before immunodetection of LAT1-GFP. We found LAT1-GFP to be more efficiently internalized in PMA-treated than in solvent-treated cells (Fig. 1C,D). We next examined whether PMA-induced internalization of LAT1-GFP leads to its degradation. As in previous experiments, before adding PMA, tetracycline was removed from the medium for 40 hours to arrest LAT1-GFP synthesis. Immunoblots revealed that LAT1-GFP turnover was much faster upon PMA treatment, and the same result was observed for the endogenous LAT1 (Fig. 1E,F). Taken together, these results show that LAT1 is internalized and degraded in PMA-treated cells.

### PMA triggers LAT1 ubiquitylation via Nedd4-2

**Figure 2:**
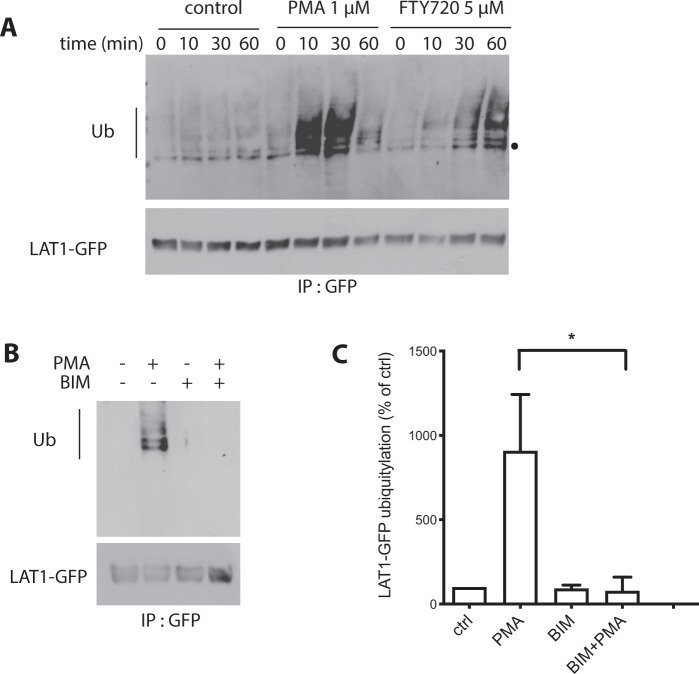
PMA promotes LAT1 ubiquitylation via PKC. (**A)** Cells of the stable T-REx HeLa line expressing LAT1-GFP were incubated with DMSO (control), 1 μM PMA, or 5 μM FTY720 for the indicated times. LAT1-GFP immunoprecipitates were probed with anti-Ub and anti-GFP antibodies. The dot indicates an unspecific band. **(B)** Cells of the stable T-REx HeLa line expressing LAT1-GFP were incubated with DMSO (control) or 1 μM PMA for 30 min at 37 °C, with or without pre-incubation with 1 μM BIM for 30 min. LAT1-GFP immunoprecipitates were probed with anti-Ub and anti-GFP antibodies. **(C)** Quantification of the immunodetected signals in three independent experiments performed as in B. Data are presented as percentages of untreated control and error bars represent standard deviations. Unpaired t-test, *p < 0.05 (PMA-treated compared with PMA and BIM-treated).

We next examined whether induced endocytosis of LAT1 requires its ubiquitylation. The HeLa T-Rex cells in which LAT1-GFP was induced were treated with PMA, and the transporter was pulled down with antibodies against GFP. After migration in a gel, the immunoprecipitates were probed with an antibody against Ub. The experiments were carried out with several batches of an anti-GFP immunoprecipitation kit, and we noticed that depending on the batch used, a nonspecific band was detected or not by the anti-Ub antibody. The experiments nevertheless revealed highly reproducible ubiquitylation of LAT1-GFP in response to PMA (Figs 2A–C, S1). The ubiquitylated forms were readily detected 10 minutes after treatment and were not present in cells in which LAT1-GFP was not induced by tetracycline (Fig. S1). LAT1-GFP was also ubiquitylated in response to FTY720, but the signal corresponding to the modification was less intense and not readily detectable until 30 minutes after treatment (Fig. 2A). The ubiquitylated LAT1-GFP forms detected upon PMA or FTY720 addition typically migrated as several bands and sometimes also as a smear, suggesting that LAT1 is polyubiquitylated and/or that several of its lysines are modified with Ub. Finally, PMA-induced ubiquitylation of LAT1-GFP was not observed in cells having been pre-incubated for 30 minutes with the PKC inhibitor BIM (bisindolylmaleimide I) (Fig. 2B,C). This confirms that PMA promotes LAT1 ubiquitylation through stimulation of PKC, as previously illustrated for several other transporters^13,16,43^. In conclusion, LAT1 undergoes efficient ubiquitylation and endocytosis upon stimulation of PKC by PMA. LAT1 is also ubiquitylated in response to FTY720, but the modification is less pronounced and occurs more slowly, consistently with our observation that FTY720 induces LAT1 endocytosis less efficiently than PMA.

**Figure 3:**
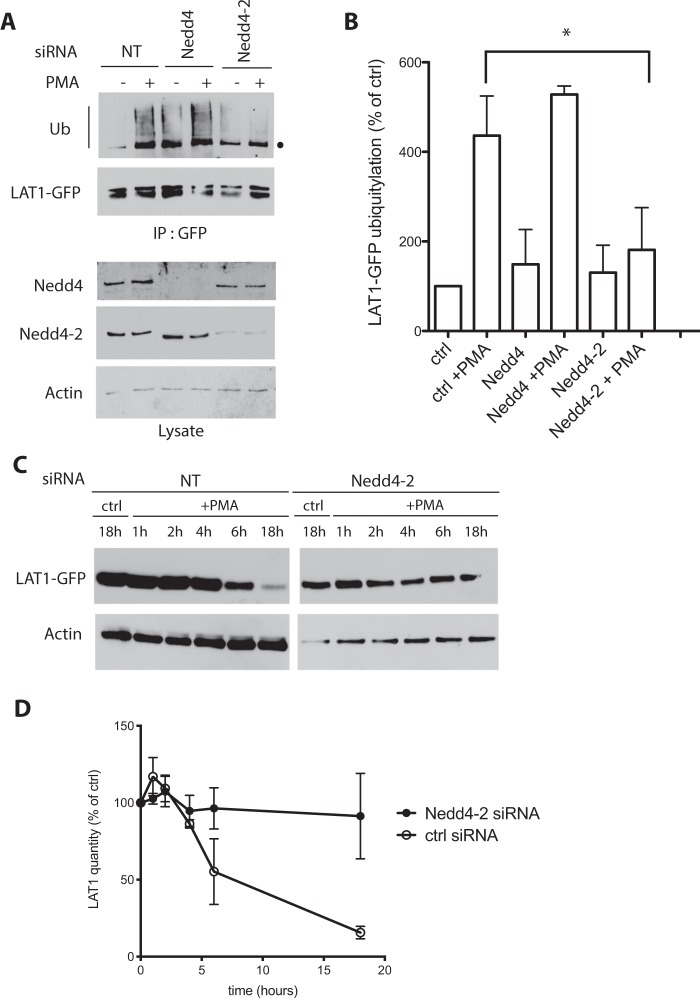
PMA-induced LAT1 ubiquitylation involves Nedd4-2. (**A**) Cells of the stable T-REx HeLa line expressing LAT1-GFP were transfected twice with control (NT), Nedd4, or Nedd4-2 siRNAs. Then cells were incubated with DMSO (control) or 1 μM PMA for 30 min. LAT1-GFP immunoprecipitates were prepared and probed with anti-Ub and anti-GFP antibodies. Total cell extracts were probed with anti-Nedd4, anti-Nedd4-2 and anti-actin antibodies. The dot indicates an unspecific band. **(B)** Quantification of the immunodetected signals in 3 independent experiments performed as in A. Data are presented as percentages of untreated control and error bars represent standard deviations. Unpaired t-test, *p < 0.05. **(C)** Cells of the stable T-REx HeLa line expressing LAT1-GFP were transfected twice with control (NT), Nedd4, or Nedd4-2 siRNAs and incubated with DMSO (control) or 1 μM PMA for the indicated times. Cell extracts were immunoblotted with anti-GFP and with anti-actin antibodies. **(D)** Quantification of the signals in immunoblots performed as in C. The graph shows the mean of three independent experiments. The error bars represent standard deviations.

Several cases of PMA-induced transporter ubiquitylation have been reported previously, and these appear most often to depend on the Nedd4 or Nedd4-2 Ub ligase^12,13,15,16^. Furthermore, a previous study has shown that siRNAs targeting Nedd4-2 impair the ∼2-fold reduction of LAT1 activity and protein level observed in human primary trophoblasts upon mTORC1 inhibition^46^. These observations prompted us to test the role of Nedd4 and Nedd4-2 in PMA-induced LAT1 ubiquitylation. We treated HeLa cells expressing LAT1-GFP with siRNAs targeting one or the other Ub ligase and observed the expected reduction of each enzyme’s expression (Fig. 3A). Upon PMA addition to cells treated with scrambled or Nedd4 siRNAs, LAT1 was ubiquitylated. In cells treated with Nedd4-2 siRNAs, LAT1 ubiquitylation was strongly reduced (Fig. 3A,B). Finally, increased turnover of LAT1-GFP in PMA-treated cells was impaired upon treatment with siRNAs targeting Nedd4-2 but not with scrambled siRNAs (Fig. 3C,D). The Nedd4-2 Ub ligase is thus required for LAT1 ubiquitylation in response to PMA and this modification promotes increased turnover of the transporter.

### Systematic mutagenesis of the N- and C-terminal tails of LAT1

**Figure 4:**
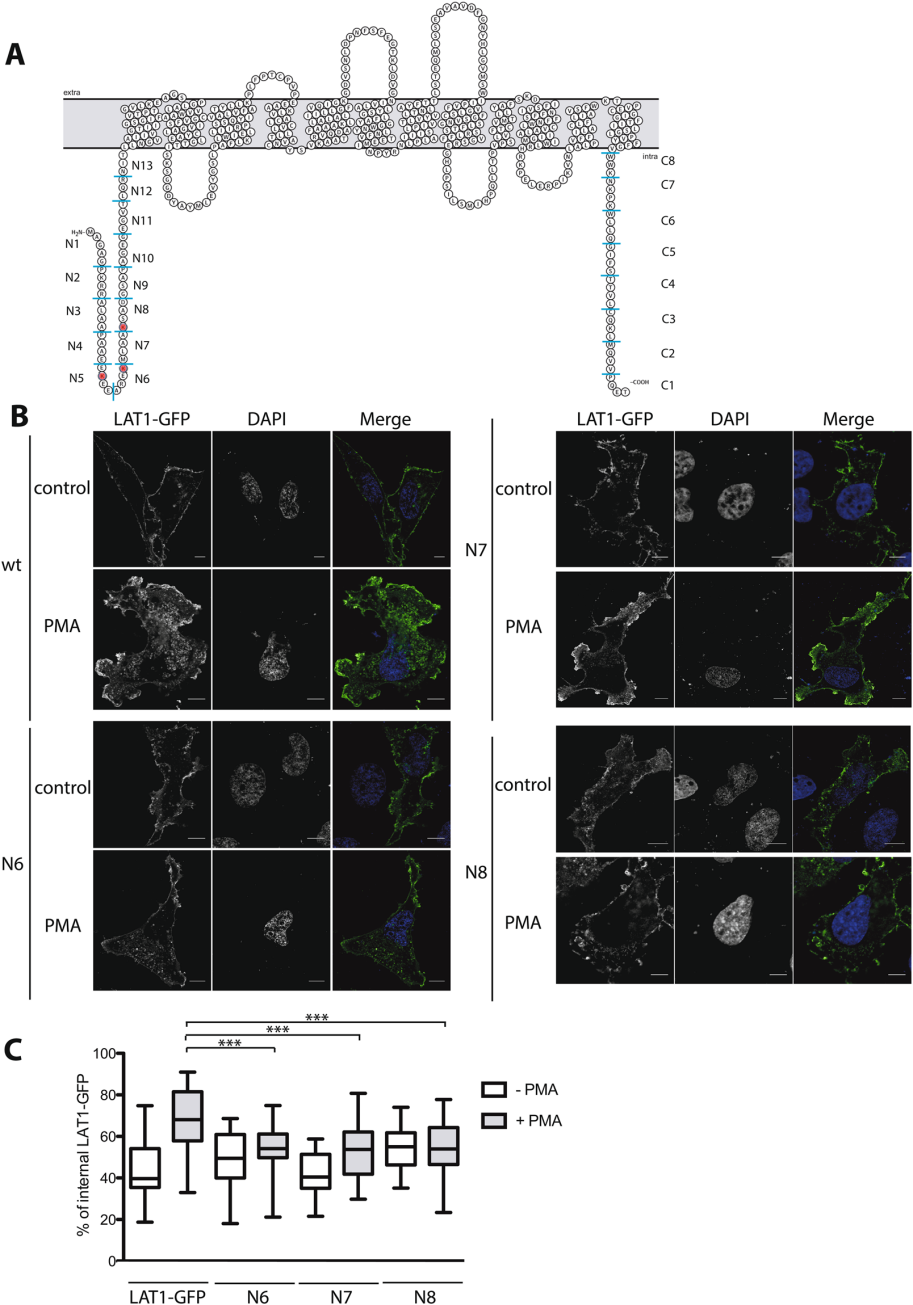
Systematic mutagenesis of LAT1 cytosolic tails identifies N-terminal residues important for PMA-induced endocytosis. (**A)** Schematic representation of the topology of LAT1 embedded in the membrane, obtained with the Protter tool^68^. The blue lines delimit the mutagenized blocks of 3 to 4 amino acids that were replaced with alanines. The three lysines (positions 19, 25, and 30) replaced with arginines are labelled in red. **(B)** HeLa cells were transiently transfected with plasmids expressing non-mutated LAT1-GFP or the indicated mutant. Cells were incubated with ammonium (NH_4_)_2_SO_4_ (25 mM) for 18 h and incubated in the presence of DMSO (control) or PMA (1 µM) for 4 h. The cells were fixed and the localization of LAT1-GFP was examined by confocal microscopy. Scale bars, 5 μm. **(C)** Quantification of the LAT1-GFP internalization shown in B. Images of at least 30 cells from three independent experiments were examined. The graph shows the percentage of intracellular LAT1-GFP determined as detailed in Methods. One-way ANOVA, ***p < 0.001.

We next sought to identify the cytosol-facing residues of LAT1 that play an important role in PMA-induced ubiquitylation and endocytosis, including the ubiquitylation target lysines. LAT1 consists of 12 transmembrane domains flanked by N- and C-terminal tails facing the cytosol^19–22^ (Fig. 4A). We focused on the N- and C-terminal tails because previous studies have shown that the transporter residues involved in endocytosis are generally located in these regions^10,47^. The limits of these tails were initially chosen on the basis of HMMTOP algorithm^48^ predictions and LAT1 structural modelling data^38–40^. They fit well with the structure of LAT1 recently solved by cryo-electron microscopy^37^. These N- and C-terminal tails contain 4 and 3 lysines, respectively, corresponding to potential ubiquitylation sites. We thus isolated 21 LAT1-GFP mutants by Ala-scanning mutagenesis of three to four consecutive amino acids in the N- and C-termini (Fig. 4A), thus covering all residues of both tails. HeLa cells transiently transfected with the 13 N-tail and 8 C-tail LAT1-GFP mutants were then treated or not with PMA and examined by confocal microscopy. HeLa cells transfected with the non-mutated LAT1-GFP construct were used as a control. In the absence of PMA, most LAT1-GFP mutants localized properly to the plasma membrane (Fig. 4B,C). The only two exceptions were LAT1 mutants with substitutions in the C-tail residues closest to the membrane (mutants C7 and C8): both displayed lower accumulation at the plasma membrane, correlating with an increased intracellular signal indicative of impaired secretion to the cell surface (Fig. S2). In the presence of PMA, most LAT1-GFP mutants having localized properly to the plasma membrane underwent a redistribution to internal membranes similar to that observed with the non-mutated LAT1-GFP. Yet three mutants in the N- tail, namely N6, N7, and N8, displayed significantly reduced internalization in response to PMA (Fig. 4B,C). These data indicate that the LAT1 N-terminal region between residues 22 and 33, which notably includes two lysines (K25 and K30), plays an important role in PMA-induced endocytosis of LAT1.

### LAT1^K19R, K25R, K30R^ is resistant to PMA-induced ubiquitylation

**Figure 5:**
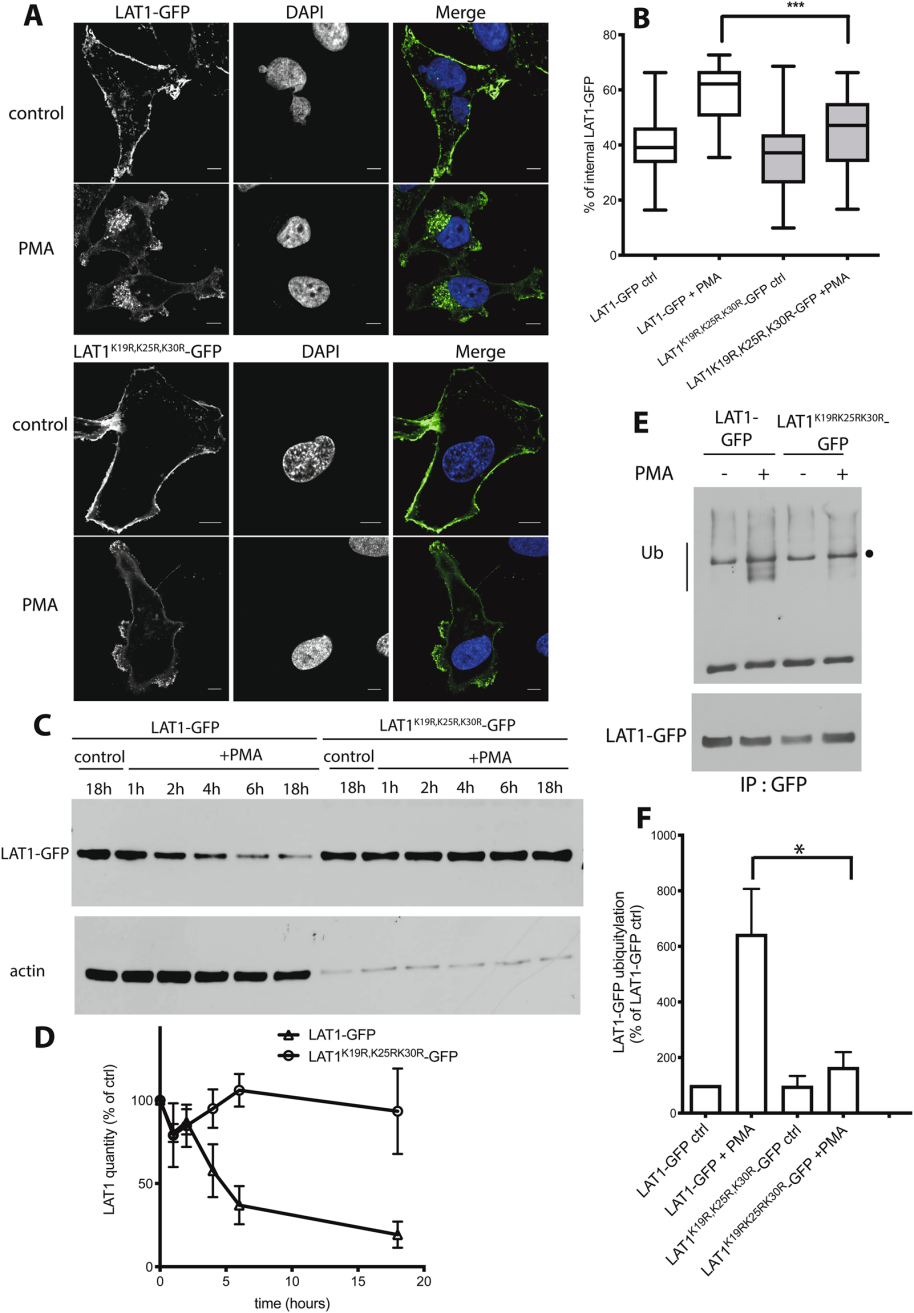
PMA-induced ubiquitylation of LAT1^K19R,K25R,K30R^ is impaired. (**A)** Cells of the stable T-REx HeLa lines expressing LAT1-GFP or LAT1^K19R,K25R,K30R^-GFP (hereafter noted as LAT1(3KR)-GFP) were incubated with 25 mM (NH_4_)_2_SO_4_ for 18 h and incubated in the presence of DMSO (control) or PMA (1 µM) for 4 h. The cells were fixed and the localization of LAT1-GFP was examined by confocal microscopy. Scale bars, 6 μm. **(B)** Quantification of the LAT1-GFP internalization shown in C. The images of at least 35 cells from three independent experiments were examined. The graph shows the percentage of intracellular immunofluorescence measured for each cell, determined as detailed in Methods. Unpaired t-test, ***p < 0.001. **(C)** Cells of the stable T-REx HeLa line expressing LAT1-GFP or LAT1(3KR)-GFP were incubated with DMSO (control) or 1 μM PMA for the indicated times. Cell extracts were immunoblotted with anti-GFP and anti-actin antibodies. **(D)** Quantification of the signals in immunoblots performed as in C. The graph shows the mean of three independent experiments. The error bars represent standard deviations. **(E)** Cells of the stable T-REx HeLa lines expressing LAT1-GFP or LAT1(3KR)-GFP were incubated with DMSO (control) or 1 μM PMA for 30 min. LAT1-GFP immunoprecipitates were prepared and probed with anti-Ub and anti-GFP antibodies. Dot indicates an unspecific band. **(F)** Quantification of the ubiquitylated LAT1-GFP signals immunodected in three independent experiments performed as in E. Data are presented as percentages with respect to the untreated control and error bars represent standard deviations. Unpaired t-test, *p < 0.05 (PMA-treated cells expressing LAT1-GFP compared to PMA-treated cells expressing LAT1(3KR)-GFP).

A previous quantitative analysis of global ubiquitylation in HeLa cells identified hundreds of ubiquitylated proteins and many precise ubiquitin attachment sites. Among these are residues K19 and K30 in the N-terminal tail of LAT1^49^. These two lysines and their neighbor K25 were later identified as targets of ubiquitylation in several other proteome-wide studies^50–53^. On the other hand, the above-described mutagenesis of the LAT1 N-terminal tail revealed that the 22-AREK-25 and 30-KSAD-33 sequences are both important for proper LAT1 endocytosis induced by PKC activation. These observations prompted us to investigate the roles of the N-terminal residues K19, K25, and K30 in PMA-triggered LAT1 ubiquitylation and endocytosis. We isolated a LAT1-GFP construct containing the triple substitution K19R-K25R-K30R, and because the conditions used to detect LAT1-GFP ubiquitylation had been set up in a stable HeLa T-Rex cell line, the mutant LAT1^K19R,K25R,K30R^, hereafter called LAT1(3KR)-GFP, was also stably expressed in these cells. After transient induction by tetracycline, LAT1(3KR)-GFP was found to be normally delivered to the plasma membrane (Fig. 5A). Upon PMA addition, while LAT1-GFP partially relocalized, as expected, to internal membranes, LAT1(3KR) was clearly retained at the cell surface (Fig. 5A,B). Furthermore, treatment of cells with PMA accelerated the turnover of LAT1-GFP, as expected, but not that of LAT1(3KR)-GFP (Fig. 5C,D). Finally, the induced LAT1-GFP and LAT1(3KR)-GFP proteins were pulled down from cell extracts, before and after PMA addition, in order to compare their ubiquitylation. LAT1-GFP showed a clear increase in ubiquitylation in response to PMA. The effect of PMA on LAT1(3KR) ubiquitylation was much lesser (Fig. 5E,F). A residual level of ubiquitylation, however, remained detectable, suggesting that other lysines in addition to K19, K25, and K30 contribute slightly to PMA-induced LAT1 ubiquitylation. In conclusion, these results demonstrate that K19, K25 and K30 are the major sites of LAT1 ubiquitylation in response to PMA. They also show that this ubiquitylation of LAT1 is crucial for its downregulation under these conditions.

## Discussion

By catalyzing the uptake of several essential amino acids including leucine, LAT1 plays an important metabolic role in many cell types, e.g. those at the placenta and the blood brain barrier^22^. LAT1 is also known as a transporter whose activity strongly influences mTORC1 activity^23–28^. Yet the mechanisms that might be involved in LAT1 regulation are not well known. In particular, those promoting its endocytosis and degradation, an inhibition mechanism commonly found for plasma membrane transporters, remain poorly characterized. In a previous work, we showed that FTY720, a sphingoid base analog with anticancer properties^42^, promotes internalization of LAT1 in HeLa cells, and we obtained evidence that the responsible signal is a reduction of mTORC1 activity caused by inhibition of nutrient transport^41^. Further investigation of the underlying mechanisms proved difficult, however, because short-term incubation of cells with FTY720 caused only partial endocytosis of LAT1, and longer-term incubation was toxic. In the present study we first defined more favorable conditions for investigating efficient LAT1 endocytosis, namely activation of PKC by the phorbol ester PMA, showing that this treatment also causes LAT1 degradation. This downregulation was observed in HeLa cells stably expressing an inducible LAT1-GFP construct, and the endogenous LAT1 underwent a similar fate. Our observations are consistent with those of a study on murine hematopoietic cells, showing internalization of 4F2hc, the type II membrane glycoprotein covalently associated with LAT1, in response to PMA addition^42^. In the same study, 4F2hc was found to be internalized upon FTY720 addition, and the effects of PMA and FTY720 were additive^42^. This is compatible with the view that these compounds stimulated LAT1 endocytosis via different pathways, a view further supported by the observation that PMA-induced stimulation of PKC provokes mTORC1 activation in various cell lines^54^, whereas FTY720 exerts the opposite effect, at least in HeLa^41^ and glioblastoma cells^55^. We next exploited the PMA treatment conditions to further explore the mechanisms responsible for induced LAT1 endocytosis. In HeLa cells, we found LAT1 to be ubiquitylated in response to PMA addition. Adding FTY720 to HeLa cells also stimulated LAT1 ubiquitylation, although the modification occurred more slowly and was less pronounced, as expected. To our knowledge, this is the first time FTY720-induced ubiquitylation of a transporter is reported in mammalian cells. This may be relevant to cancer therapy, as FTY720 and related compounds cause downregulation of several nutrient transporters in tumor cells, and as their use is envisaged as a possible strategy for starving them to death^56^.

Further analysis of LAT1 ubiquitylation upon PKC activation revealed that it is largely impaired when three close lysines in the cytosolic N-terminal tail (K19, K25 and K30) are replaced with arginines. These lysines are thus the main sites of LAT1 ubiquitylation under the tested conditions. They might be used for basal-level ubiquitylation and endocytosis of LAT1 as well, as they were also identified in systematic analyses of Ub-conjugated residues in HeLa and other cell lines not treated with PMA^49–53^. Although the ubiquitylation of LAT1 is clearly needed for its efficient endocytosis in PMA-treated cells, further experiments are needed to determine the exact roles of this modification in LAT1 membrane trafficking. For instance, while Ub could provide a signal for LAT1 sorting into endocytic vesicles, LAT1 ubiquitylation might also be important to prevent recycling of the internalized transporter to the cell surface. It is indeed well illustrated that once transporters have reached the endosomes, the Ub moieties linked to them may provide a signal for their subsequent sorting into vesicles budding into the endosomal lumen, as the endosomes mature into a multivesicular bodies (MVBs). This MVB sorting of internalized transporters, involving the complexes of the ESCRT machinery, is crucial for their final delivery into the lysosomal lumen, upon fusion of MVBs with lysosomes^2,4^.

In our study, the LAT1 lysines required for ubiquitylation were initially identified during systematic mutagenesis of the residues present in the N-terminal tail of the transporter. This mutagenesis further revealed that the MLAA sequence close to the three identified lysines is also important for PMA-induced LAT1 endocytosis. Furthermore, mutagenesis applied to the C-terminal tail revealed two neighboring sequences, close to the membrane, essential to proper secretion of LAT1 to the plasma membrane. Interestingly, these results are reminiscent of those obtained when the cytosolic residues of the yeast general amino acid permease, Gap1, were mutagenized by means of a similar Ala-scanning approach^57^. Specifically, the N-terminal tail of Gap1 includes two lysines that are ubiquitylated, and a sequence close to these residues is also essential to Gap1 ubiquitylation, at least under particular conditions^57,58^. The C-terminal tail, in contrast, plays a more important role in secretion to the plasma membrane^57^. It thus seems that human LAT1 and yeast Gap1, which belong to the same structural family of amino acid transporters (those sharing the LeuT fold), rely on similar mechanisms for controlling their intracellular trafficking.

Our study also reveals that PMA-induced LAT1 ubiquitylation depends on the Nedd4-2 Ub ligase. This provides another similarity to yeast Gap1, which is ubiquitylated by the homologous Rsp5 enzyme^47^. These observations agree with those of another study, showing reduced LAT1 ubiquitylation in human primary trophoblasts upon depletion of Nedd4-2^46^. The authors hypothesize that ubiquitylated LAT1 is targeted to the proteasome for degradation. As discussed above, we rather think that ubiquitylated LAT1 is targeted to the lysosomes for degradation. This view is supported by our observation that LAT1 accumulates in intracellular compartments in cells are treated with NH_4_^+^, an inhibitor of lysosomal degradation.

The Ub ligases of the Nedd4 family and the homologous yeast Rsp5 protein interact with target proteins via their WW domains recognizing PY motifs^9^. For instance, Nedd4-2 interacts directly with the PY motifs of subunits the ENaC Na^+^ channel^8^. In yeast, the WW domains of Rsp5 bind to PY motifs exposed by several adaptors, including proteins of the α-arrestin family, and these proteins target different sets of permeases^59,60^. Furthermore, the physiological regulation of permease ubiquitylation often proceeds through direct control of these α-arrestin adaptors.^3,4,58^. Sequences displaying all the properties expected of binding sites for α-arrestins have been found in the N-terminal tails of several permeases, close to ubiquitylated lysines^58,61,62^. How Nedd4-2 targets LAT1 in PMA-treated HeLa cells remains unknown. As the cytosolic regions of LAT1 do not contain any canonical PY motif, it could be that Nedd4-2 interacts with LAT1 via an adaptor protein. If so, the N-terminal sequence (MLAA) close to the ubiquitylation-target lysines of LAT1, important for proper PMA-induced LAT1 internalization, might be a binding site for that adaptor, which might be one of the six arrestin-domain-containing proteins (ARRDC1-5 and TXNIP) homologous to the yeast α-arrestins. Although these proteins remain very partially characterized, ARRDC3 has been implicated in the Nedd4-2-dependent ubiquitylation and degradation of the β2-adrenergic receptor^63^, and TXNIP contributes importantly to endocytosis of the Glut1 and Glut4 glucose transporters^17,18^. Additional work is needed to determine whether LAT1 ubiquitylation and endocytosis involve these AARDC proteins or possibly another adaptor.

Nedd4-2 is reported to mediate PMA-induced ubiquitylation of several other transporters and, as in the case of LAT1, this modification has been found to trigger their endocytosis and accelerate their degradation. These proteins include the dopamine transporter (DAT)^12^, glutamate transporter 1 (GLT-1)^13^, and cationic amino acid transporter 1 (CAT-1)^16^. It thus seems that upon activation of PKC by PMA, Nedd4-2 promotes ubiquitylation of multiple transporters in various cell types, thereby causing their endocytosis. According to another report, the neurotoxic effect of manganese is characterized by downregulation of the ASCT2 and SNAT3 glutamine transporters, likely via Nedd4-2-mediated ubiquitylation. In this case, it was shown that manganese increases the activity of several PKC isoforms needed for downregulation of these glutamine uptake systems^15^. In the future, it will be interesting to elucidate how PKC activation leads to Nedd4-2-mediated transporter ubiquitylation. A plausible mechanism is modulation of the interaction properties of Nedd4-2 by phosphorylation^64^. For instance, García-Tardón *et al*. (2012) report that Nedd4-2 is phosphorylated upon PMA addition to COS cells, this modification correlating with its enhanced interaction with the GLT-1 transporter^13^. Similarly, Nedd4-2 phosphorylation by AMPK enhances its binding to ENaC and hence reduces epithelial Na^+^ transport^65^. In contrast, phosphorylation of Nedd4-2 by different kinases, at residues located between the WW domains, inhibits its interaction with the ENaC subunits. In this case, inhibition of the interaction is due to binding of 14-3-3 proteins to phosphorylated Nedd4-2^64^. In yeast, permease ubiquitylation has been shown to be triggered by direct control of α-arrestins or by transport-catalysis-coupled conformational changes^3,4,58,61^. According to another report, however, Gap1 ubiquitylation is also controlled by phosphorylation of Rsp5^66^.

In conclusion, we report for the first time a regulatory mechanism, of potential physiological relevance, affecting LAT1: endocytosis and degradation, triggered by Nedd4-2-mediated ubiquitylation of three lysines in the transporter’s cytosolic N-terminal tail. Future work will determine the importance of this mechanism in normal and tumor cells, where LAT1 plays an important metabolic role.

## Methods

### Human cell culture and transfection

Cells were grown at 37 °C under an atmosphere of 5% CO_2_. HeLa cells were grown in Dulbecco’s modified Eagle’s medium (DMEM) with 10% fetal bovine serum and 1% penicillin/streptomycin. HeLa T-REx cells stably expressing LAT1-GFP^41^ were grown in MEM Glutamax supplemented with 10% fetal bovine serum, 1% penicillin/streptomycin, 5 µg/ml blasticidin and 200 μg/ml zeocin. Expression of LAT1-GFP in the T-Rex-inducing system was induced by supplementing the medium with 1 μg/mL tetracycline for 24 hours.

Cells were transfected with Plasmid DNA or siRNA using lipofectamine 2000 according to the manufacturer’s instructions. Nedd4-1 (4390824), Nedd4-2 (4392420), and control siRNA (4390843) were purchased from Ambion. Cells were transfected with siRNA 48 h and 72 h before performing an experiment.

### Construction of plasmids

Plasmid pCB001, expressing LAT1-GFP under the control of the tetracycline-regulated promoter and able to replicate also in yeast, has been described previously^41^. The 21 LAT1-GFP mutants were constructed by homologous recombination in yeast between two partially overlapping PCR fragments and the linearized vector pCB001.

### Generation of a stable cell line

A stable cell line expressing the LAT1^K19R,K25R,K30R^-GFP mutant was generated as previously described^41^. T-REx HeLa cells were transfected with the plasmid expressing LAT1^K19R,K25R,K30R^-GFP. Forty-eight hours after transfection, cells were transferred for 3 weeks to a selection medium including 5 µg/ml blasticidin and 200 μg/ml zeocin. Individual clones were isolated, expanded, and grown in MEM Glutamax containing 10% FBS, blasticidin, zeocin, and 1% penicillin/streptomycin. After expansion, single colonies were tested for LAT1^K19R,K25R,K30R^-GFP expression by addition of 1 μg/ml tetracycline for 24 h and subsequent analysis by western blotting and fluorescence microscopy.

### Immunofluorescence analysis of T-REx HeLa cells

10^5^ Cells were seeded on coverslips in 24-well plates. Expression of LAT1-GFP was induced by treatment for 24 h with 1 µg/ml tetracycline before washing the cells and growing them for 40 h in a tetracycline-free medium and incubated them with 25 mM (NH_4_)_2_SO_4_ during 18 h before adding PMA or DMSO for 4 h. Cells were fixed as indicated in^41^. DAPI was used for nuclear staining. The coverslips were mounted on microscope slides and the cells were viewed with an inverted confocal microscope (Zeiss LSM 710 with a 63 × /1.4 objective). Images were processed with ImageJ and Adobe Illustrator. The intracellular immunofluorescence was determined for each cell with this formula: (intracellular pixel intensity – mean background pixel intensity x area of selected cells)/(total cellular pixel intensity - mean background pixel intensity x area of selected cells) x100. The intracellular areas were manually defined and pixel intensities were measured using ImageJ.

### Immunoprecipitation of LAT1-GFP

Experiments were based on a previously published method^67^. Cells were seeded on 6-well plates and grown to 80–90% confluency. Expression of LAT1-GFP was induced by treatment for 24 h with 1 µg/ml tetracycline before removing the antibiotic and leaving the cells for a further 40 h in tetracycline-free medium. Cells were treated with 1 μM PMA for the indicated time. After washing with CMF-PBS, the cells were lysed with ice-cold lysis buffer and the cell lysates were cleared by centrifuging for 15 min at 16,000xg. The supernatant was incubated with anti-GFP microbeads (μMACS GFP isolation kit; Miltenyi biotec) for 2 h at 4 °C. The beads were washed four times with 200 μl lysis buffer, and LAT1-GFP was eluted from the beads according to the manufacturer’s instructions.

### Protein extracts, western blotting

Cells were lysed with cold NP40 buffer (150 mM NaCl, 1% NP40, 50 mM Tris-HCl pH 8) supplemented with protease inhibitor cocktail (Roche, n°04693159001). An equal volume of 2x loading buffer (500 mM Tris Base, 50 mM Tris HCl pH 6.8, 2 mM EDTA, 2% SDS, 10% glycerol, 0.01% bromophenol blue, and 2% β-mercaptoethanol) was added to the cell lysates and the solution was heated for 10 min at 42 °C. After transfer to a nitrocellulose membrane (Schleicher and Schuell), the proteins were probed with anti-GFP (Roche, catalog nbr 11814460001, 1/10000×), anti-LAT1 (Abcam, ab85226, 1/2500) anti-actin (Santa-Cruz, sc-47778 1/5000), anti-ubiquitin P4D1 (Santa-Cruz, sc-8017, 1/2000), anti-Nedd4 C5F5 (Cell Signaling, 3607 S, 1/5000), or anti-Neddd4-2 antibody (Abcam, ab131167, 1/5000). Primary antibodies were detected with horseradish peroxidase-conjugated anti-mouse or anti-rabbit immunoglobulin G secondary antibody (GE Healthcare, 1/10000×). Bound antibodies were revealed by chemiluminescence (Roche, 12015196001). Non-cropped versions of the immunoblots are presented in the Supplementary Information file (Fig. S2).

### Cell-surface biotinylation

Surface biotinylation assays were performed as previously described^41^. Cells were seeded on 6-well plates at a cell density of 5 × 10^5^ cells per well. LAT1-GFP expression was induced by a 24-h treatment with 1 µg/ml tetracycline before removing the antibiotic and leaving the cells for a further 40 h in tetracycline-free medium. Cells were washed twice with PBS containing 1 mM MgCl and 0.1 mM CaCl_2_ and incubated with sulfo-NHS-biotin for 30 min at 4 °C. The cells were then incubated for various times either at 4 °C, to block membrane trafficking, or at 37 °C, in the presence or absence of PMA, to allow internalization to occur. For removal of cell-surface biotin, the cells were incubated with Mesna buffer (50 mM Tris pH 8.8, 100 mM NaCl, 50 mM Mesna, and 0.2% fetal bovine serum) for 40 min to reduce disulfide bonds. Then the cells were incubated for 20 minutes in quenching buffer (50 mM Tris pH 8.8, 100 mM NaCl, 50 mM iodoacetamide, 0.2% fetal bovine serum), then washed twice with PBS. The cells were lysed with NP40 buffer and biotinylated proteins were purified by affinity chromatography with streptavidin. LAT1-GFP was then detected by immunoblotting with anti-GFP antibody.

### Statistical analysis

Statistical analyses were performed with Prism 6 software. Unpaired t-test or one-way analysis of variance (ANOVA) with a Dunnet’s post hoc test were used as specified. Error bars on graphs show standard deviations.

## Supplementary information

Supplementary Information

## Data Availability

All data generated and analyzed during this study are included in this published article (and its Supplementary Information File). Additional data corresponding to negative results are available from the corresponding author on request. The biological materials and data sets generated during the current study are available from the corresponding author on reasonable request.
