# Supplementary material for: Ubiquitylation and endocytosis of the human LAT1/SLC7A5 amino acid transporter
Source: Sci Rep. 2019 Nov 14;9:16760. doi: 10.1038/s41598-019-53065-w (PMC6856120; doi:10.1038/s41598-019-53065-w)
Supplement: Supplementary file 1 — Supplementary Information [file 41598_2019_53065_MOESM1_ESM.pdf]

Supplementary information

Ubiquitylation and endocytosis of the human LAT1/SLC7A5 amino acid transporter

Barthelemy Céline and Bruno André

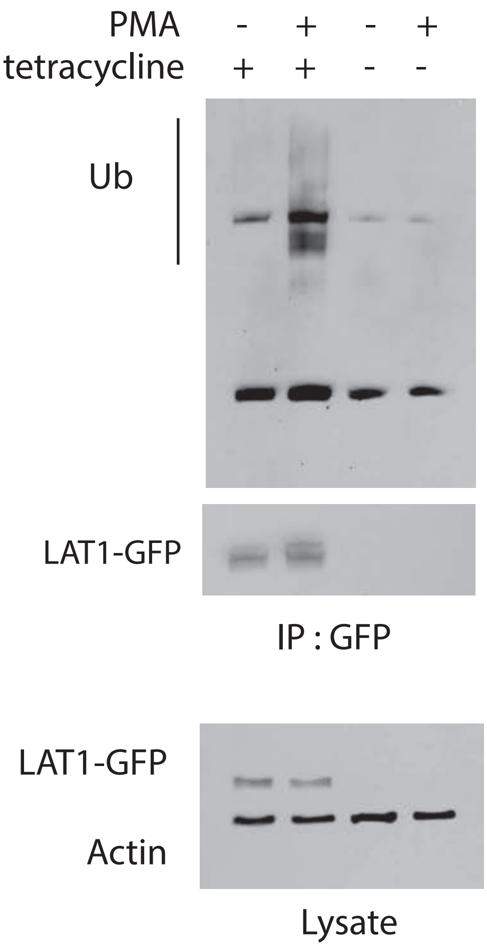

Figure S1. PMA promotes LAT1 ubiquitylation. Cells of the stable T-REx HeLa line expressing LAT1-GFP (+ tetracycline) or not (- tetracycline) were incubated with DMSO (control) or 1 µM PMA for 30 min at 37°C. Proteins were purified by IP and immunoprecipitates were probed with anti-Ub and anti-GFP antibodies. Total extracts were probes with anti-GFP and anti-actin antibodies.

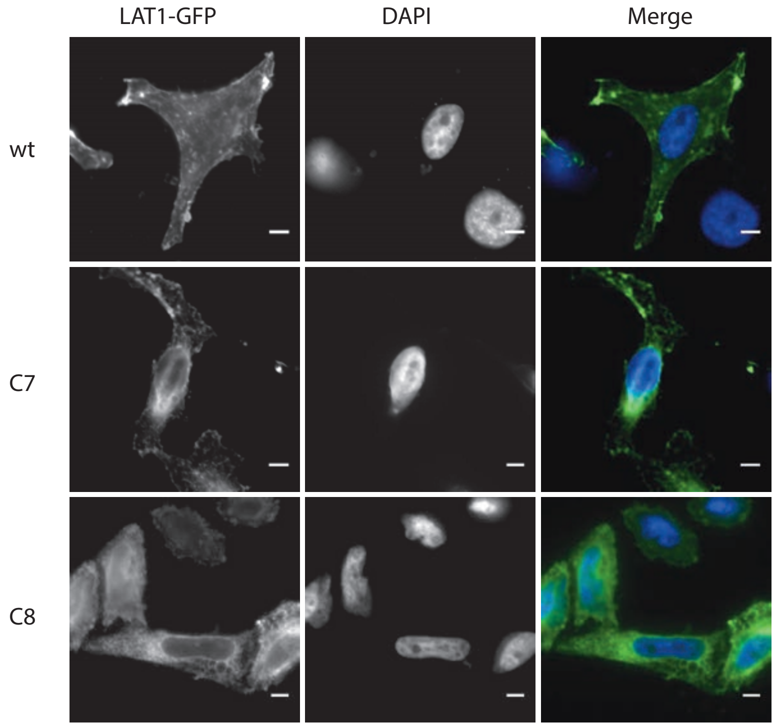

Figure S2. Systematic mutagenesis of LAT1 cytosolic tails identifies two C-terminal mutants with impaired secretion to the cell surface. HeLa cells were transiently transfected with plasmids expressing non-mutated LAT1-GFP or the indicated mutant. The cells were fixed and the localization of LAT1-GFP was examined by confocal microscopy. Scale bars, 3 μm.

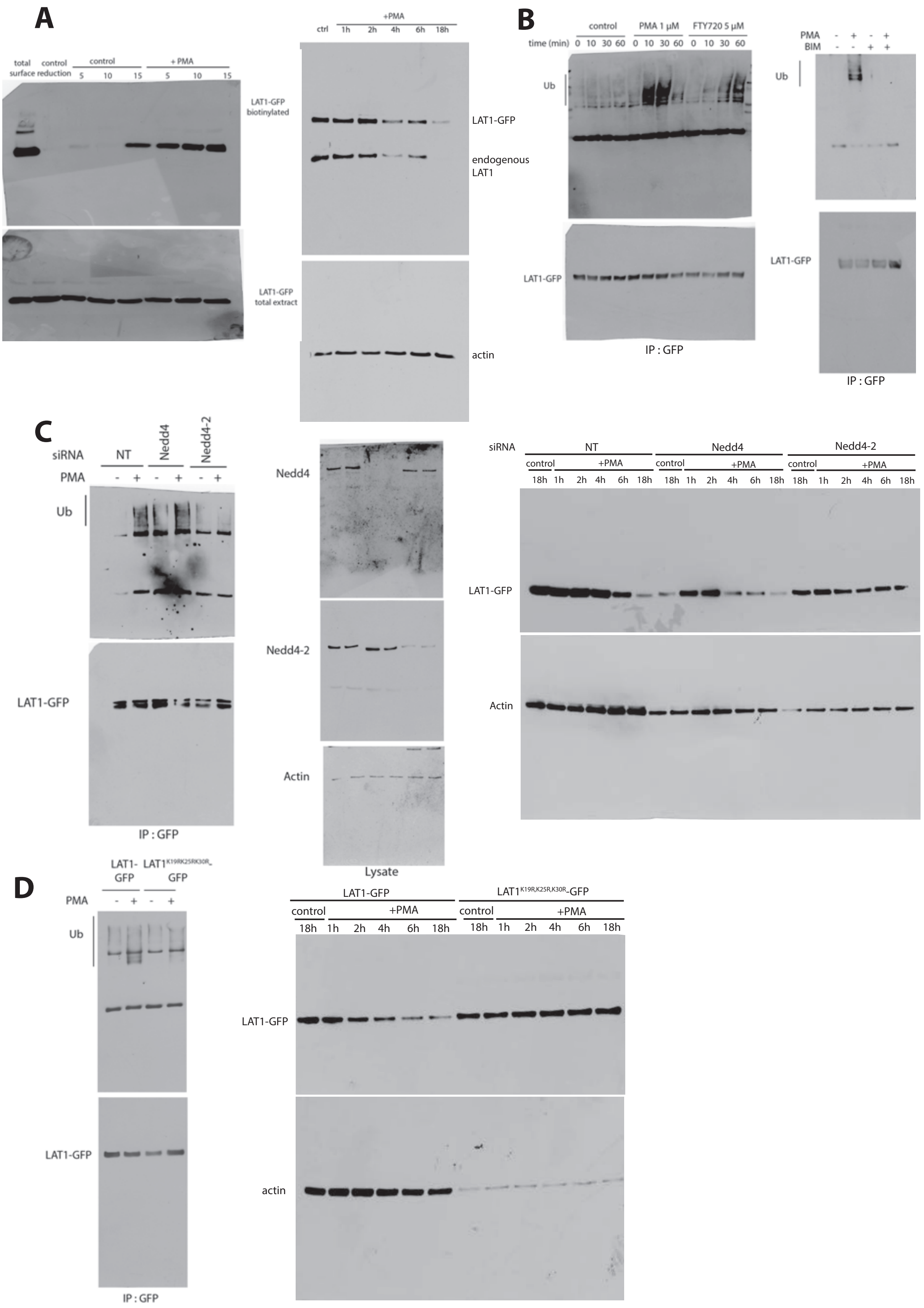

Figure S3. Full-length blots. Non-cropped versions of the blots presented in Figure 1 (A), Figure 2 (B), Figure 3 (C), and Figure 5 (D).
